# Variations in the Bacterial, Fungal, and Protist Communities and Their Interactions Within Sediment Affected by the Benthic Organism, Snail *Bellamya purificata*

**DOI:** 10.3390/microorganisms12122550

**Published:** 2024-12-11

**Authors:** Yiran Hou, Yiyun Zhang, Rui Jia, Linjun Zhou, Bing Li, Jian Zhu

**Affiliations:** 1 Key Laboratory of Integrated Rice-Fish Farming Ecology, Ministry of Agriculture and Rural Affairs, Freshwater Fisheries Research Center, Chinese Academy of Fishery Sciences, Wuxi 214081, China; houyr@ffrc.cn (Y.H.); jiar@ffrc.cn (R.J.); 2 Wuxi Fisheries College, Nanjing Agricultural University, Wuxi 214081, China; zyyffrc@hotmail.com (Y.Z.); zhoulinjun@ffrc.cn (L.Z.)

**Keywords:** *Bellamya purificata*, sediment, microbial community, bacterial, fungal, protist communities

## Abstract

In aquatic benthic environments, benthic organisms have been found to regulate important biogeochemical characteristics and perform key ecosystem functions. To further explore the ecological impact of the snail *Bellamya purificata*’s, presence on the benthic environment, we employed high-throughput sequencing technology to investigate its effects on the bacterial, fungal, and protist communities in sediment and their intrinsic interactions. Our findings revealed that *B. purificata*’s presence significantly enhanced the diversity and evenness of the fungal community while simultaneously decreasing the diversity and richness of the protist community, and it also altered the composition and relative abundance of the dominant phyla across the bacterial, fungal, and protist communities. The snail *B. purificata* considerably altered the co-occurrence networks of the microbial communities, particularly by enhancing the intrinsic complexity of the protist community and by strengthening the interconnections among the protist, bacterial, and fungal communities. Notably, the proportions of specialists within the sediment bacterial, fungal, and protist communities declined due to the snail *B. purificata*. Its presence also notably expanded the habitat niche breadth for sediment bacteria and protists. In terms of community assembly, *B. purificata* shifted the fungal community assembly from being dominated by stochastic processes to being dominated by deterministic processes, whereas the protist community assembly shifted from deterministic processes to being dominated by stochastic processes. The mainly altered ecological processes in the fungal and protist assemblies were drift and homogenizing selection. Additionally, the presence of *B. purificata* resulted in a notable reduction in the sediment ON level and a significant increase in the ammonia, FA, and EN concentrations. Sediment properties, particularly FA and nitrate, were strongly correlated with microbial communities and were key contributors to changes in microbial community dynamics. These research findings not only broadened our understanding of the ecological impacts of *B. purificata* on benthic microbial communities but also highlighted its substantial potential in enhancing microbial community stability.

## 1. Introduction

Benthic fauna have been found to regulate important biogeochemical properties and enable crucial ecosystem functions in aquatic benthic environments [1]. Benthic fauna can affect the physical and chemical properties of sediment and accelerate the material circulation and transformation at the sediment–water interface through motion, ingestion, breathing, excretion, and burrowing, etc. [2,3,4,5,6,7]. Different benthic species exhibit different bioturbation activities, such as sediment particle reworking and bioirrigation activities, which can cause varying bioturbation effects [8].

Microbial communities are important and non-negligible indicators in both natural and artificial aquatic environments as they reflect the ecological status of the ecosystem [9,10,11,12,13,14]. Microorganisms, including bacteria, fungi, and protists, are key drivers in aquatic ecosystems, playing vital roles in ecosystem functions and biogeochemical processes [15,16,17,18,19,20,21]. Bioturbation derived from the benthic fauna can affect the benthic microbial community, thereby influencing microbial functions and microbially-mediated biogeochemical processes [22,23]. Furthermore, the interactions between bacteria, fungi, and protists are notably complex and diverse in aquatic ecosystems. Protists and fungi can break down complex organic materials that are resistant to bacterial decomposition, thus impacting bacterial community growth [24,25,26,27]. Heterotrophic protists can consume bacteria, thereby altering bacterial community structure, while autotrophic protists can influence these communities through provision of shelter and nutrients [28,29]. The impact of bioturbation by benthic animals on microbial communities will inevitably alter the communities of bacteria, fungi, and protists in the sediment, as well as their interactions. Consequently, elucidating the changes in the communities of bacteria, fungi, and protists in sediments and their intrinsic relationships is essential for understanding the crucial role of benthic animals to aquatic ecosystems.

Snail species, as typical benthic fauna, play a vital role in the benthic environment within aquatic ecosystems. Various freshwater snail species have been widely used for water purification, eutrophication restoration, and environmental biomonitoring in both natural and artificial aquatic ecosystems [30,31,32,33,34]. *Bellamya purificata* is a highly representative freshwater snail, widely distributed across ponds, lakes, reservoirs, rivers, and other aquatic ecosystems in China [35,36]. Snail *B. purificata* prefers to inhabit silt and ingest organic debris and algae in its surroundings [35,36]. Previous research has demonstrated that *Bellamya* sp. plays an important role in water purification and materials cycling at the sediment–water interface during aquaculture production and these snails alter the bacterial and fungal communities in the aquaculture environment [37,38,39,40]. However, most of these studies have not primarily focused on *B. purificata*’s ecological effect, nor have they excluded the effects of feed inputs when assessing the ecological effects of *B. purificata*’s presence. Overall, comprehensive studies on the effects of *B. purificata* on bacterial, fungal, and protist communities in sediment and their interrelationships remain markedly limited.

Hence, our study aimed at investigating the effects of *B. purificata*’s presence on the communities of bacteria, fungi, and protists in sediment and their inherent interactions. We analyzed and compared the diversity, composition, co-occurrence network structure, environmental adaptability, and assembly processes of these communities with or without the *B. purificata* presence condition. We also evaluated the correlations of these metrics with the physicochemical properties of sediment, exploring the potential mechanisms by which their presence affected bacterial, fungal, and protist communities and their inherent interactions. Our research will provide more in-depth insights into the ecological effects of this typical benthic animal.

## 2. Materials and Methods

### 2.1. Indoor Simulation Experiment Design

The experiment was carried out at the laboratory of the Freshwater Fisheries Research Center, Chinese Academy of Fishery Sciences (FFRC, CAFS) (120°19′6.888″ E, 31°29′55.716″ N; Wuxi, China). The snail *Bellamya purificata* and the experimental sediment were obtained from ponds located in the aquaculture facility of FFRC (119°55′56.06″ E, 31°18′41.69″ N; Wuxi, China). Prior to the experiment, the snails were kept in glass tanks for 14 d to acclimate to the laboratory conditions. The experimental sediments were dried in the sun, ground to a powder in a mortar to pass through a 100-µm mesh sieve, and mixed evenly to ensure consistency and homogeneity before use [41,42,43]. In the experiment, a treatment group containing *B. purificata* snails (BG group) and a control group without snails (CG group) were established, each comprising six replicates. Twelve glass tanks, measuring 78 cm × 39 cm × 48 cm, were utilized and were layered with a 5.0-cm depth of sediment at the bottom. Prior to the experiment, the sediment in each tank was allowed to settle for 14 days. After acclimation, healthy snails *B. purificata*, each weighing about 3 g, were randomly and equally allocated to six glass tanks designated for the BG group, with 26 snails per tank, resulting in a stocking density of approximately 262.82 g/m^2^. The fish commercial feed for pond aquaculture, sourced from Zhejiang Haida Feed Co., Ltd. (Shaoxing, China), was finely crushed and sieved through a 100-µm mesh. Every day at 16:00, the snails were fed with this crushed and sieved commercial feed at a rate corresponding to 2% of their total weight. Before feeding, it was necessary to ensure that the feed was thoroughly mixed with water and then evenly poured into the tank. Although the CG group did not have snails, it still needed to receive the same quantity of feed as the BG group, and the feeding method for the CG group was identical to that of the BG group. In addition to the snail *B. purificata* farming and presence, strict consistency was maintained in the experimental conditions between the CG and BG groups. The room temperature was maintained at 25 ± 0.5 °C. The experiment was conducted under natural lighting conditions. The water used in the experiment was fully aerated tap water, and one-third of the tap water was changed daily. The entire experimental period lasted for 90 days.

### 2.2. Sediment Sample Collection

At the end of the experiment, nine sampling points were randomly selected in each glass tank to collect surface sediment within the 0–1 cm range. The nine sediment samples collected from the same tank were uniformly mixed into one sample. The collected samples were divided into two parts; one part was freeze-dried at −65 °C using the CHRIST LYO Alpha 1-4 LD plus (Martin Christ Gefriertrocknungsanlagen GmbH, Osterode am Harz, Germany), then ground and stored at −20 °C for the determination of total nitrogen (TN), organic nitrogen (ON), fixed ammonium (FA), exchangeable nitrogen (EN), ammonia, nitrate, and nitrite. The other part was preserved directly at −80 °C for the analysis and determination of bacterial, fungal, and protist communities.

### 2.3. Sediment Properties Determination

The TN, ammonia, nitrate, and nitrite concentrations in sediment were determined according to Chinese national standards by various spectrophotometric methods, as detailed in Table 1. The FA level in sediment was measured by Nessler’s reagent spectrophotometry method according to Li et al. [44] (Table 1). The EN content in sediment was calculated as the sum of ammonia, nitrate, and nitrite concentrations, whereas ON level was then determined by subtracting the sum of EN and FA from the TN content.

### 2.4. DNA Extraction, Sequencing, and Data Processing

Microbial DNA, including that of bacteria, fungi, and protists, was extracted from sediment using the E.Z.N.A.^®^ Soil DNA Kit (Omega Bio-tek, Inc., Norcross, GA, USA). Specific primers were used to amplify the 16S rRNA V3-V4 regions for bacteria, ITS1-ITS2 regions for fungi, and 18S rRNA V4 regions for protists (Table 2) [46,47,48]. The amplified products were quantified and purified, followed by library preparation using the NEXTFLEX^®^ Rapid DNA-Seq Kit (Bioo Scientific Corporation., Austin, TX, USA). Sequencing was carried out on the Illumina NovaSeq PE250 platform.

Quality control of the raw sequencing reads was performed using FASTP (version 0.23.4), and the assembly was conducted with FLASH (version 0.23.4), setting the minimum overlap length at 10 bp and the allowable mismatch error rate at 2% [49,50]. Subsequently, the retained reads were deduplicated and analyzed using the Divisive Amplicon Denoising Algorithm 2 (DADA2) within Quantitative Insights into Microbial Ecology 2 (QIIME 2) to identify insertions/deletions and substitution mutations, and was then classified as Amplicon Sequence Variants (ASVs) [51]. Paired-end reads were trimmed and filtered, allowing a maximum of two expected errors per read (maxEE ≤ 2). The ASVs for bacteria, fungi, and protists were, respectively, classified and identified using the Silva (SSU132) database, the UNITE database, and the Protist Ribosomal Reference database (PR2) [52,53,54].

### 2.5. Statistical Analysis

The Shannon, Simpson, Chao 1, and Pielou_J indices were employed to evaluate the α-diversity of bacterial, fungal, and protist communities within the sediment samples. Differences in the structures of these communities across the BG and CG groups were analyzed through Principal Coordinates Analysis (PCoA) using the weighted Bray–Curtis distance. The permutational multivariate analysis of variance (PERMANOVA) test was used to further confirm the statistical discrepancy in these communities between groups demonstrated by the PCoA. The Wilcoxon rank–sum test was conducted to assess variation in community composition at the phylum level between the two groups, with significance set at *p* < 0.05. Based on Spearman correlation matrices derived from 16S rRNA, ITS, and 18S rRNA sequencing data (Spearman r > 0.8 and *p* < 0.05), co-occurrence networks were established for bacterial, fungal, and protist communities to evaluate the intra- and inter-assemblage interactions. The breadth of habitat niches and dispersal abilities of these communities were quantified to gauge their environmental adaptability according to Xiong et al. [55]. Furthermore, the generalist and specialist species within the bacterial, fungal, and protist communities were identified, respectively, using the Levins’ niche breadth index [55]. Moreover, the relationship between microbial communities and sediment physicochemical properties was assessed using distance-based redundancy analysis (db-RDA), and the relative impact of these properties on microbial community dynamics was explored using an aggregated boosted tree (ABT). Null model analysis combined with the neutral community model was employed to identify the respective influences of deterministic and stochastic processes on the bacterial community assembly within paddy water [56,57]. During the null model analysis, the β-nearest taxon index (βNTI) and the Raup–Crick metric (RC) were used as criteria to determine the ecological processes shaping the water’s bacterial community [58].

## 3. Results

### 3.1. Diversity and Composition of Sediment Bacterial, Fungal, and Protist Communities

**Figure 1:**
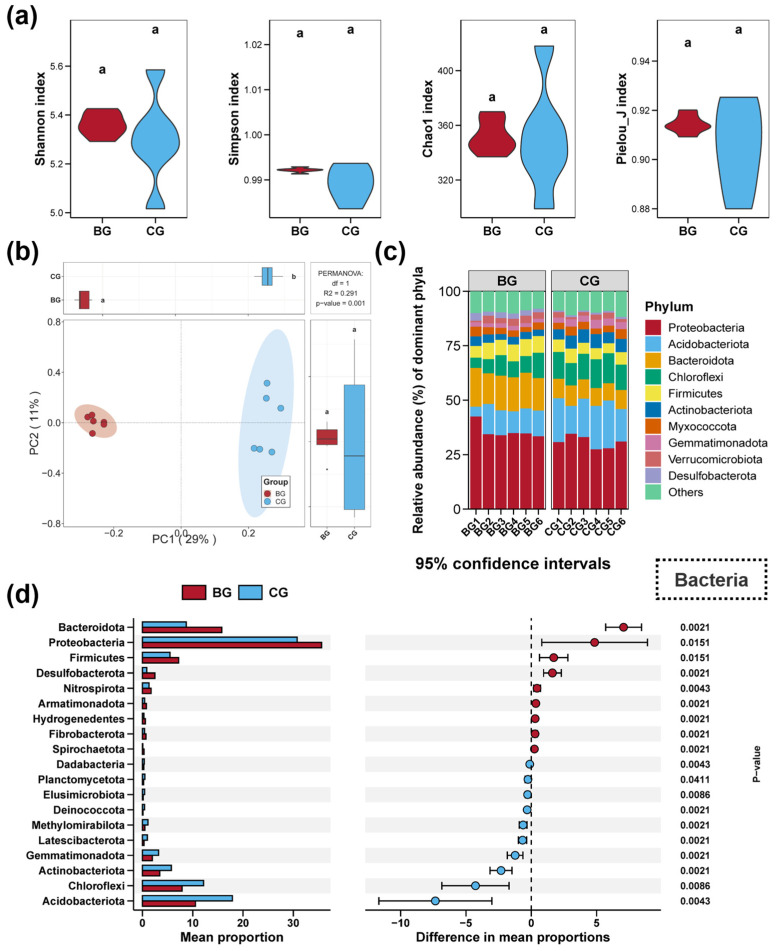
Impacts of snail *Bellamya purificata* presence on the diversity and composition of sediment bacterial community. (**a**) Differences in the bacterial Shannon, Simpson, Chao1, and Pielou_J induced within sediment between the BG and CG groups. (**b**) Differences in sediment bacterial communities demonstrated by PCoA (Principal Coordinates Analysis). (**c**) Composition of the top ten bacterial phyla in terms of relative abundance. (**d**) Bacterial phyla with considerable differences between the BG and CG groups. Distinct lowercase letters marked in the violin plots or box plots signify statistically significant differences (*p* < 0.05).

Impacts of snail *B. purificata* presence on the diversity and composition of the sediment bacterial community are illustrated in Figure 1. There were no significant differences between the BG group and the CG group in terms of the Shannon, Simpson, Chao1, and Pielou_J indices of the sediment bacterial community (Figure 1a, *p* > 0.05). PCoA and PERMANOVA analyses revealed remarkable disparities in the sediment bacterial communities of these groups, with each group demonstrating distinct clustering visually (Figure 1b, *p* < 0.05). The ten most dominant phyla in terms of relative abundance across all the sediment samples included Proteobacteria, Acidobacteriota, Bacteroidota, Chloroflexi, Firmicutes, Actinobacteriota, Myxococcota, Gemmatimonadota, Verrucomicrobiota, and Desulfobacterota (Figure 1c). Notably, the BG group substantially altered eight of these bacterial phyla (Figure 1d, *p* < 0.05). Relative to the CG group, the BG group obviously promoted the Proteobacteria, Bacteroidota, Firmicutes, and Desulfobacterota in sediment, whereas the Acidobacteriota, Chloroflexi, Actinobacteriota, and Gemmatimonadota were considerably reduced (Figure 1d, *p* < 0.05).

**Figure 2:**
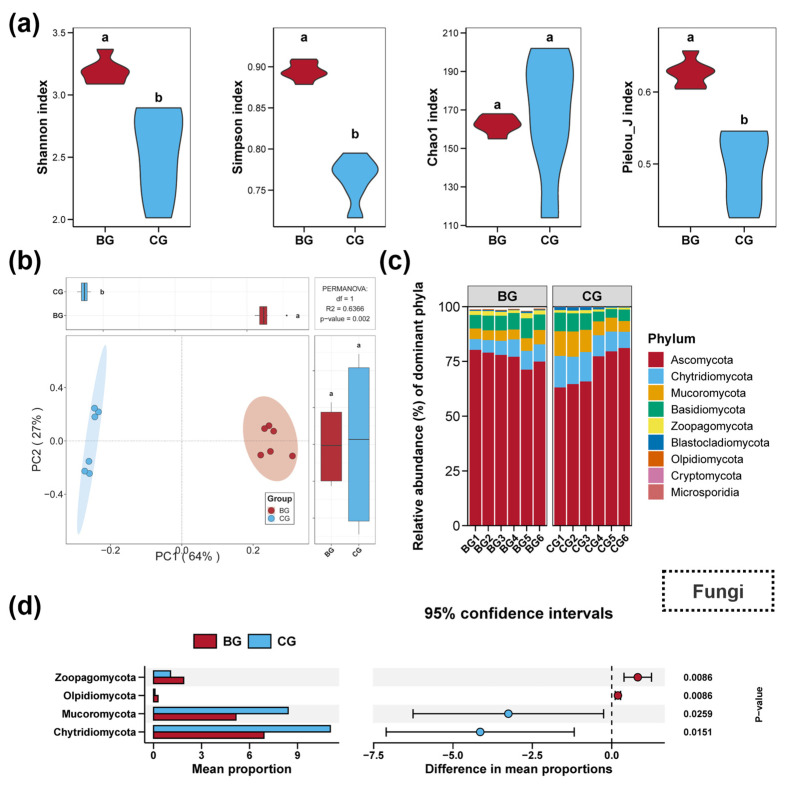
Impacts of snail *Bellamya purificata* presence on the diversity and composition of sediment fungal community. (**a**) Differences in the fungal Shannon, Simpson, Chao1, and Pielou_J induced within sediment between the BG and CG groups. (**b**) Differences in sediment fungal communities demonstrated by PCoA (Principal Coordinates Analysis). (**c**) Composition of the top ten fungal phyla in terms of relative abundance. (**d**) Fungal phyla with considerable differences between the BG and CG groups. Distinct lowercase letters marked in the violin plots or box plots signify statistically significant differences (*p* < 0.05).

Impacts of snail *B. purificata* presence on the diversity and composition of the sediment fungal community are revealed in Figure 2. The BG group significantly improved the Shannon, Simpson, and Pielou_J diversity indices of the sediment fungal community (Figure 2a, *p* < 0.05), but no obvious differences in Chao1 were observed between the BG and CG groups (Figure 2a, *p* > 0.05). The results of PCoA and PERMANOVA tests exhibited notable differences in the sediment fungal communities between BG and CG groups, with each group clustering separately visually (Figure 2b, *p* < 0.05). The fungal community within all the sediment samples comprised nine phyla, listed in order of relative abundance: Ascomycota, Chytridiomycota, Mucoromycota, Basidiomycota, Zoopagomycota, Blastocladiomycota, Olpidiomycota, Cryptomycota, and Microsporidia (Figure 2c). Among these, the BG group exhibited a significant increase in the relative abundance of Zoopagomycota and Olpidiomycota compared to the CG group, while the abundances of Mucoromycota and Chytridiomycota were decreased (Figure 2d, *p* < 0.05).

**Figure 3:**
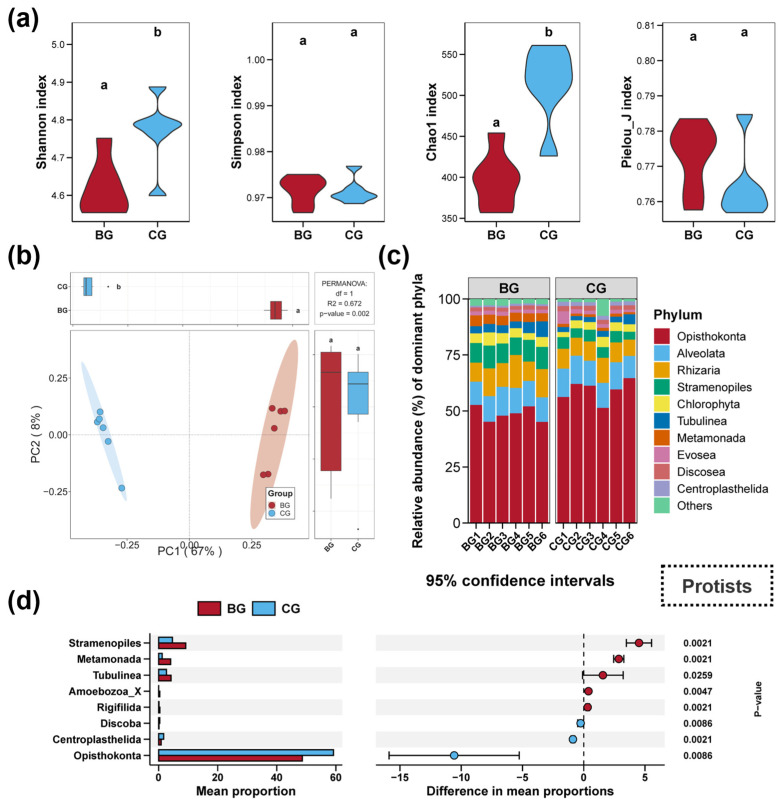
Impacts of snail *Bellamya purificata* presence on the diversity and composition of sediment protist community. (**a**) Differences in the protist Shannon, Simpson, Chao1, and Pielou_J induced within sediment between the BG and CG groups. (**b**) Differences in sediment protist communities demonstrated by PCoA (Principal Coordinates Analysis). (**c**) Composition of the top ten protist phyla in terms of relative abundance. (**d**) Protist phyla with considerable differences between the BG and CG groups. Distinct lowercase letters marked in the violin plots or box plots signify statistically significant differences (*p* < 0.05).

Impacts of snail *B. purificata* presence on the diversity and composition of sediment protist community are revealed in Figure 3. The BG group significantly reduced the Shannon and Chao1 diversity indices of the sediment protist communities (Figure 3a, *p* < 0.05). However, no significant differences were observed in the Simpson and Pielou_J indices between the BG and CG groups (Figure 3a, *p* > 0.05). The PCoA and PERMANOVA analyses revealed marked differences between the sediment protist communities of the BG and CG groups, with distinct clustering observed visually for each group (Figure 3a, *p* < 0.05). In the sediment samples, the community composition at the phylum level predominantly consisted of the following phyla, ranked by relative abundance: Opisthokonta, Alveolata, Rhizaria, Stramenopiles, Chlorophyta, Tubulinea, Metamonada, Evosea, Discosea, and Centroplasthelida (Figure 3c). Specifically, compared to the CG group, the BG group notably promoted Stramenopiles, Metamonada, and Tubulinea in the sediment, while decreasing Opisthokonta (Figure 3d, *p* < 0.05).

### 3.2. Co-Occurrence Network Patterns of Sediment Bacterial, Fungal, and Protist Communities

**Figure 4:**
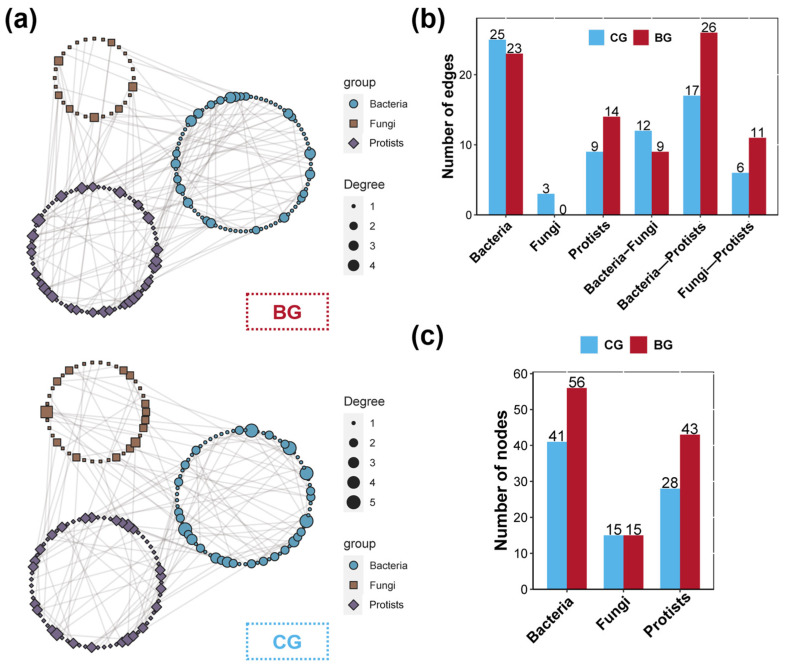
Alterations in the interactions within and between bacteria, fungi, and protist communities across the BG and CG groups. (**a**) Co-occurrence networks representing the interactions within and between bacteria, fungi, and protist communities across the BG and CG groups. (**b**) The number of edges in the co-occurrence networks for bacteria, fungi, and protist communities across the BG and CG groups. (**c**) The number of nodes in the co-occurrence networks for bacteria, fungi, and protist communities across the BG and CG groups.

Microbial co-occurrence networks were established to explore the interactions within and between communities of bacteria, fungi, and protists, as shown in Figure 4a. For the BG group, the edge number in the co-occurrence networks of bacterial, fungal, and protist communities in the sediment were 23, 0, and 14, respectively; for the CG group, they were 25, 3, and 9, respectively (Figure 4b). The node number for these communities in the BG group were 56 for bacteria, 15 for fungi, and 43 for protists; whereas, in the CG group, the counts were 41, 15, and 28, respectively (Figure 4c). Regarding inter-community interactions, the bacterial–fungal co-occurrence networks contained 9 edges for the BG group and 12 for the CG group; bacteria–protist co-occurrence networks included 26 edges for the BG group and 17 for the CG group, and the fungal–protist networks had 11 for the BG group and 6 for the CG group (Figure 4b).

### 3.3. Environmental Adaption and Habitat Niche Breadth of Sediment Bacterial, Fungal, and Protist Communities

**Figure 5:**
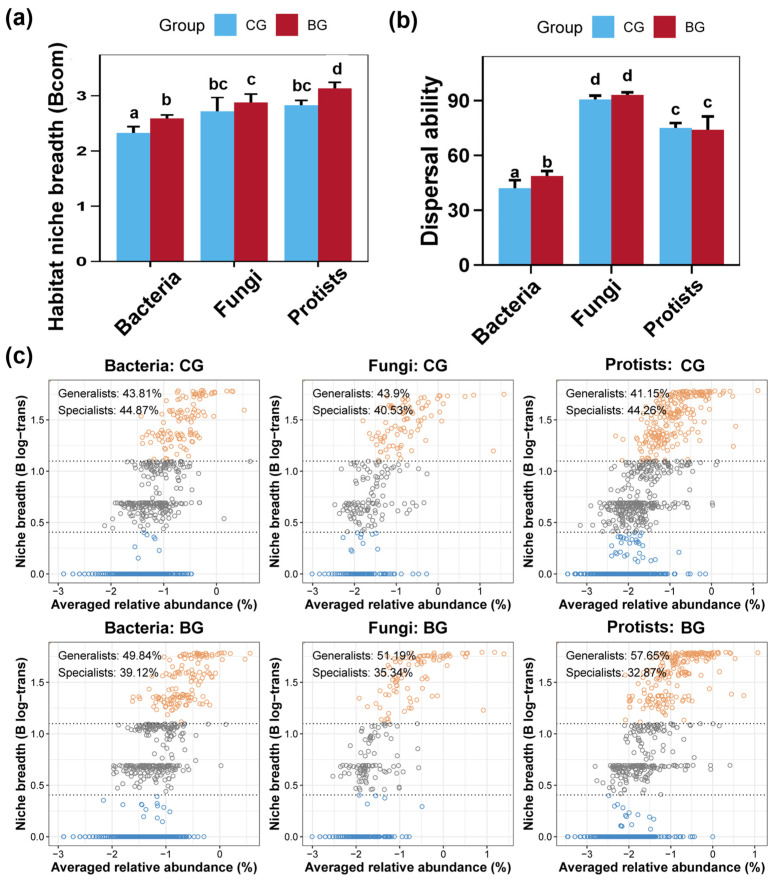
Impacts of snail *Bellamya purificata* presence on the environmental adaption and habitat niche breadth of sediment bacterial, fungal, and protist communities. (**a**) The habitat niche breadth of bacterial, fungal, and protist communities and their differences between BG and CG groups. (**b**) The dispersal ability of bacterial, fungal, and protist communities and their differences between BG and CG groups. (**c**) Proportion of generalist and specialist bacteria, fungi, and protist in the sediment of the BG and CG groups. Distinct lowercase letters marked in bar charts or box plots indicate remarkable differences between the BG and CG groups (*p* < 0.05).

In this study, overall, the habitat niche breadth of bacterial community in the sediment was smaller than that of the fungal community, which in turn was smaller than that of the protist community (Figure 5a). Conversely, the dispersal ability was highest within the sediment’s fungal community, followed by the protist and bacterial community (Figure 5b). Compared to the CG group, the BG group substantially broadened the habitat niche breadth of the sediment bacterial and protist communities (Figure 5a, *p* < 0.05), but did not affect the fungal community (Figure 5a, *p* > 0.05). Additionally, the BG group significantly enhanced the dispersal capability of the sediment bacterial community without significant impacts on the fungal and protist communities (Figure 5a). Microbes were categorized as generalists or specialists based on their habitat niche breadth. Within the BG group, specialists and generalists in the sediment bacterial community were 39.12% and 49.84% respectively, while in the CG group, specialists and generalists were 44.87% and 43.81%. For the sediment fungal community, the BG group had specialist and generalist proportions of 35.34% and 51.19%, compared to 40.53% and 43.90% in the CG group. In terms of the sediment protist communities, the BG group had 32.87% specialists and 57.65% generalists, contrasting with 42.26% and 41.15% in the CG group, respectively.

### 3.4. Assembly Processes of Sediment Bacterial, Fungal, and Protist Communities

**Figure 6:**
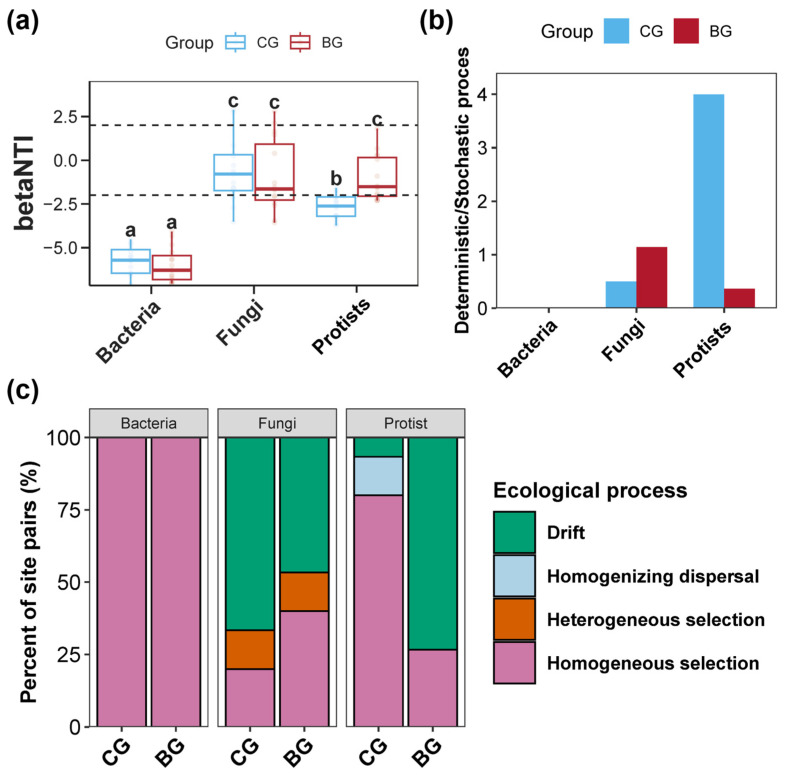
Impacts of snail *Bellamya purificata* presence on the assembly processes of sediment bacterial, fungal, and protist communities. (**a**) Differences in the β-nearest taxon index (βNTI) value of the sediment bacterial, fungal, and protist assembly between the BG and CG groups. (**b**) Differences in the proportion of deterministic to stochastic processes in the assembly processes shaping bacterial, fungal, and protist communities between the BG and CG groups. (**c**) Proportions of ecological processes in the assembly processes of sediment bacterial, fungal, and protist communities across BG and CG groups. Distinct lowercase letters marked on the box plot indicate obvious differences between the BG and CG groups (*p* < 0.05).

Variations in the assembly processes of bacterial, fungal, and protist communities within the sediment of the BG and CG groups are shown in Figure 6. The Null model analysis suggested that the BG group obviously elevated the βNTI values for the protist community within the sediment relative to the CG group, although this increase was not mirrored in the βNTI values for the bacterial and fungal communities between the two groups (Figure 6a, *p* < 0.05). Furthermore, the average βNTI value for the sediment bacterial community was below −5.0, indicating a predominantly deterministic process shaping the community (Figure 6a). In addition, the BG group markedly reduced the proportion of the deterministic process to the stochastic process in the protist community assembly, favoring a shift towards stochastic predominance (Figure 6b). Further specification of the ecological processes involved in deterministic and stochastic processes revealed that deterministic processes in the bacterial communities were characterized by homogeneous selection (Figure 6c). In the sediment fungal community, deterministic processes included both heterogeneous and homogeneous selection, with stochastic processes being drift (Figure 6c). For the protist community, homogeneous selection characterized the deterministic processes, while stochastic processes involved drift and homogenizing dispersal (Figure 6c). Notably, in the BG group, the proportion of drift in the fungal community assembly processes was reduced, augmenting the role of homogeneous selection (Figure 6c). Conversely, in the protist community, there was an increase in the proportion of drift coupled with a reduction in homogeneous selection (Figure 6c).

### 3.5. Associations Between Sediment Properties and Bacterial, Fungal, and Protist Communities

**Figure 7:**
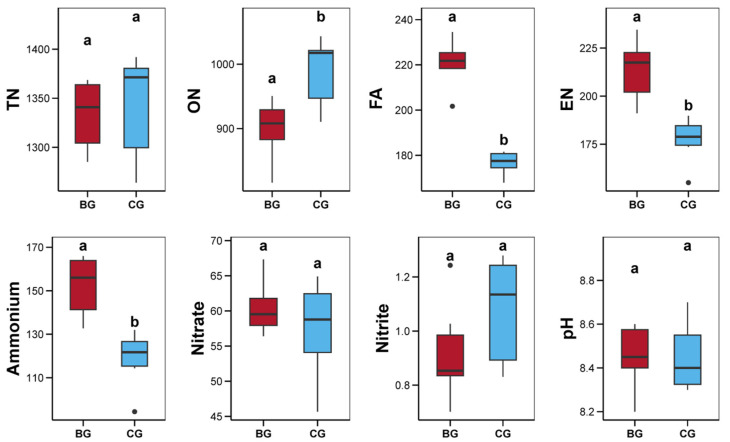
Impacts of snail *Bellamya purificata* presence on the sediment properties including total nitrogen (TN, µg/g), organic nitrogen (ON, µg/g), fixed ammonium (FA, µg/g), exchangeable nitrogen (EN, µg/g), ammonium (µg/g), nitrate (µg/g), and nitrite (µg/g). Distinct lowercase letters marked on the box plot indicate obvious differences between the BG and CG groups (*p* < 0.05).

**Figure 8:**
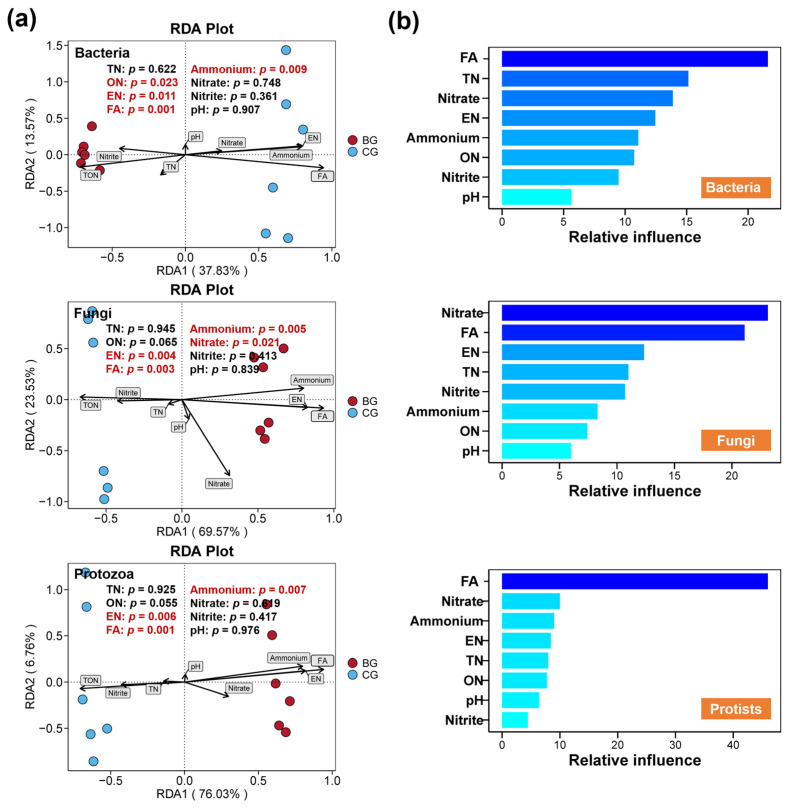
Associations between sediment properties and bacterial, fungal, and protist communities. (**a**) Correlations of the sediment properties with the bacterial, fungal, and protist communities assessed by distance-based redundancy analysis (db-RDA) across BG and CG groups. (**b**) Relative contributions of the soil properties to the changes in bacterial, fungal, and protist communities evaluated by aggregated boosted tree (ABT).

As illustrated in Figure 7, comparisons between the BG and CG group revealed that the BG group considerably altered the physicochemical properties of the sediment, notably increasing the FA, EN, and ammonium concentrations, while reducing the ON level in sediment. Concurrently, these physicochemical properties exhibited notable correlations with bacterial, fungal, and protist communities (Figure 8a, *p* < 0.05). Particularly, the levels of FA, EN, and ammonium were positively correlated with these microbial communities in the BG group, but negatively correlated with those in the CG group (Figure 8a, *p* < 0.05). Additionally, the sediment’s ON content was considerably correlated with the bacterial community, and the nitrate level was remarkably correlated with the fungal community (Figure 8a, *p* < 0.05). According to ABT analysis, FA emerged as the primary contributor to changes in the communities of bacteria, fungi, and protists, with nitrate also playing a substantial role in altering the fungal community (Figure 8b).

## 4. Discussion

### 4.1. Snail B. purificata Presence Altered the Diversity and Composition of Sediment Bacterial, Fungal, and Protist Communities

Prior research has revealed that benthic animals can impact the composition, diversity, metabolism, and function of benthic bacterial communities [41,59,60,61]. For our study, the snail *B. purificata* remarkably affected the diversity and composition of bacterial, fungal, and protist communities in the sediment. Changes in the Shannon, Simpson, Chao1, and Pielou_J indices suggested that *B. purificata* presence considerably enhanced the diversity and evenness of the fungal community, but reduced the diversity and richness of the protist community. Meanwhile, snail *B. purificata* presence significantly promoted the dominant bacterial phyla Proteobacteria, Firmicutes, and Bacteroidota, while reducing the relative abundances of Acidobacteriota, Chloroflexi, and Actinobacteriota in the sediment. Previous research has reported that bioturbation caused by benthic animals altered the relative abundance of Proteobacteria, Bacteroidota, Acidobacteriota, and Actinobacteriota [62,63,64]. Bacterial phyla Proteobacteria, Firmicutes, Bacteroidota, Acidobacteriota, Chloroflexi, and Actinobacteriota are all involved in biogeochemical cycles, serving as the primary drivers in the carbon, nitrogen, phosphorus, or sulfur cycles [65,66,67,68,69,70]. Variations in their relative abundance also demonstrated the impact of snail *B. purificata* presence on the ecological functions of the sediment’s bacterial community.

Studies exploring the impact of benthic animals on benthic fungal and protist communities remains limited. Engel, et al. [71] documented that the lugworm *Arenicola marina* could significantly influence the composition and abundance of protist communities in intertidal zones. Wu, et al. [72] discovered that bioturbation by crabs could substantially alter the composition and functionality of intertidal fungal communities. For our study, *B. purificata* noticeably increased the fungus Zoopagomycota and the protist Stramenopiles, while reducing the fungi Chytridiomycota and Mucoromycota, along with the protist Opisthokonta. Despite belonging to one of the earliest lineages of fungi, species within the phylum Zoopagomycota remain both understudied and incompletely characterized, with numerous lineages continuing to be poorly understood [73]. Moreover, the fungus Chytridiomycota has been proven to participate in the decomposition of difficult-to-degrade organic materials such as lignin [74]; the protist Stramenopiles acts as both consumer and producer in aquatic environments, playing a significant role in the carbon and minerals cycles [75]. Thus, changes in the composition of fungal and protist communities also reflect the impact of the snail *B. purificata*’s presence on the ecological functions of these communities.

### 4.2. Snail B. purificata Presence Altered the Co-Occurrence Networks for Sediment Bacterial, Fungal, and Protist Communities and Their Intrinsic Interaction Relationships

Microbial co-occurrence networks reveal the complex interactions within communities, where the number of nodes and edges characterizes the complexity and connectivity strength of microbial communities [76,77]. A higher number of edges and nodes reflects an increase in ecological traits shared among microbes as well as functional redundancy, which helps microbial communities to withstand external pressures and maintain stability in the face of environmental changes [78,79]. In this study, using a threshold where variations in the number of edges and nodes exceeded 50%, we found that the snail *B. purificata* considerably enhanced the intrinsic complexity of the protist community, as well as strengthening the internal relationships between the protist community and the bacterial and fungal communities [80,81]. This finding implied that the *B. purificata* presence strongly promoted the stability of the protist community and its interactions with bacterial and fungal communities in the sediment, which might be beneficial to the entire microbial community’s stability.

We further analyzed the environmental adaptability and sensitivity to provide ecological evidence for the stability of microbial communities. Microorganisms are categorized as generalists or specialists based on the breadth of their ecological niche [82,83]. Generalists contribute to ecosystem stability with wide distribution, whereas specialists, more vulnerable and sensitive to environmental changes, indicate a strong environmental sensitivity for the community when they are present in a high proportion [84,85,86,87]. In the present study, the proportions of specialists within the sediment bacterial, fungal, and protist communities in the BG group were 39.12%, 35.34%, and 32.87%, respectively, which were lower than those in the CG group at 44.87%, 40.53%, and 42.26%, respectively. This further confirmed that the snail *B. purificata* reduces the sensitivity of sediment microbial communities to environmental changes and increases their stability. Additionally, the habitat niche breadth reflects the stability characteristics of microbial communities, where a narrower habitat niche breadth indicates weaker metabolic plasticity and environmental adaptability of microbial populations [88]. The significant expansion of habitat niche breadth for sediment bacteria and protist communities caused by the snail *B. purificata* might also suggest its contribution to enhancing the stability of microbial communities.

### 4.3. Snail B. purificata Presence Altered the Assembly Processes Shaping Sediment Bacterial, Fungal, and Protist Communities

The ecological effect caused by the snail *B. purificata* induced dramatic changes in the assembly processes of the fungal and protist communities in the sediment. The mechanisms that shape the composition and diversity within and among multispecies communities are generally known as ecological processes [89]. Selection, dispersal, drift, and speciation are the main ecological mechanisms that facilitate microbial community assembly [89,90]. Selection, driven by physical, chemical, and biological forces, can modify the relative abundance of species based on their survival and reproductive abilities [89]. Meanwhile, dispersal and drift can affect the diversity within and between multispecies communities [89]. In our study, the snail *B. purificata* remarkably affected the processes of drift and homogenizing selection, thereby impacting the assembly of fungal and protist communities. Notable variations in the composition and diversity of fungal and protist communities also confirmed the profound influence of *B. purificata* on these ecological processes. Similar to previous studies, the simultaneous occurrence of lower community diversity and the dominance of drift in community assembly processes was observed in the sediment protist community affected by the snail *B. purificata* [91,92].

Species occurrence patterns are impacted by environmental variables either directly or indirectly, including environmental filtering and interference in species competition outcomes [93,94]. Environmental factors are closely associated with the assembly processes of microbial communities; shifts in these factors can alter the relative contributions of different ecological processes involved in assembly [95,96,97,98]. Therefore, in our study, the influence of *B. purificata* presence on the assembly processes of fungal and protist communities were likely caused by alterations in the sediment physicochemical properties. Intriguingly, these ecological effects on the assembly processes of fungal and protist communities were diametrically opposite; *B. purificata* shifted the fungal community assembly from being dominated by stochastic processes to being dominated by deterministic processes, whereas the protist community assembly shifted from deterministic processes to being dominated by stochastic processes. Previous studies have observed that minor changes in environmental variables induce stochastic assembly of the microbial community, while major changes lead to deterministic assembly [96,97,98,99]. However, our findings indicated that under the same environmental changes, the assembly mechanisms of distinctly different types of microbial communities, such as fungi and protists, can exhibit varied changes or responses.

### 4.4. Alterations in Sediment Properties and Their Associations with Bacterial, Fungal, and Protist Communities

Bioturbation activities derived from the benthic organisms can alter the physical structure and chemical properties of sediments, accelerate the exchange of materials at the sediment–water interface, and affect the biogeochemical processes at this interface [2,3,4,5,6,7]. Previous studies have shown that the snail *B. purificata*’s bioturbation can effectively promote the degradation of organic matter in sediments, reducing the content of organic matter therein [100]. Similarly, in the present study, *B. purificata* presence resulted in a notable reduction in the sediment ON level and a significant increase in the ammonia, FA, and EN concentrations, indicating an accelerated degradation of organic nitrogen into ammonia, subsequently increasing the sediment FA and EN.

Environmental conditions and microbial communities exhibit a complex intrinsic relationship [101]. Soil microbes not only participate in nutrient cycling and the transformation of organic materials but also directly alter the physical and chemical properties of soil through processes like biological weathering and bioprecipitation [101]. Meanwhile, variations in environmental conditions, in turn, affect the formation and activity of microbial communities, indirectly shaping the structure and diversity of microbes [101,102,103]. Nutritional environmental conditions, such as nitrogen and phosphorus, are considered in many studies to significantly impact microbial communities by altering microbial growth, abundance, and activity [104,105]. For our study, sediment properties also exhibited considerable correlations with bacterial, fungal, and protist communities, especially the FA and nitrate concentrations in sediment, which made the greatest contribution to the variations in microbial communities. Therefore, changes in sediment properties might be a primary indirect pathway through which *B. purificata* affects bacterial, fungal, and protist communities in sediment.

## 5. Conclusions

In conclusion, the snail *B. purificata* remarkably affected the bacterial, fungal, and protist communities and their interactions within sediment. Specifically, *B. purificata* presence considerably enhanced the diversity and evenness of the fungal community while reducing the diversity and richness of the protist community. Moreover, *B. purificata* also altered the composition and relative abundance of dominant phyla in bacterial, fungal, and protist communities. According to the co-occurrence network analysis, *B. purificata* notably changed the numbers of nodes and edges within bacterial, fungal, and protist co-occurrence networks, increasing the intrinsic complexity of the protist community and strengthening the interconnections between protist, bacterial, and fungal communities. It was noteworthy that the presence of *B. purificata* has led to a decrease in the proportion of specialists in the bacterial, fungal, and protist communities in the sediment, and has significantly expanded the habitat niche breadth for sediment bacteria and protists. During the microbial assembly process, the snail *B. purificata* induced a shift in fungal community assembly from being dominated by stochastic processes to being dominated by deterministic processes, while the assembly of the protist community shifted from deterministic to stochastic dominance. The mainly altered ecological processes in the fungal and protist assemblies were drift and homogenizing selection. Additionally, *B. purificata* markedly decreased the ON level in the sediment while significantly enhancing the ammonia, FA, and EN concentrations. The nutrient levels were strongly correlated with the sediment bacterial, fungal, and protist communities within sediment and played crucial roles influencing the dynamics of microbial community shifts. These research findings confirmed the substantial potential of the snail *B. purificata* in enhancing microbial community stability, effectively broadening our understanding of the ecological impacts of *B. purificata* presence on benthic microbial communities.

## Figures and Tables

**Table 1:** The determination measurements for the sediment physicochemical indicator.

Physicochemical Indicator	Measurement Methods
Total nitrogen (TN)	Modified Kjeldahl method [45]
Fixed ammonium (FA)	Nessler’s reagent spectrophotometry method [44]
Ammonia	Extraction with potassium chloride solution-spectrophotometric methods [45]
Nitrate	
Nitrite	

**Table 2:** Amplification regions and primers for bacterial, fungal, and protistan DNA.

Category	Amplification Region	Primer
Bacteria	16S rRNA V3-V4	338F (5′-ACTCCTACGGGAGGCAGCAG-3′)
		806R (5′-GGACTACHVGGGTWTCTAAT-3′)
Fungi	ITS1-ITS2	ITS1F (5′-CTTGGTCATTTAGAGGAAGTAA-3′)
		ITS2R (5′-GCTGCGTTCTTCATCGATGC-3′)
Protist	18S rRNA V4	TAReuk454FWD1 (5′-CCAGCASCYGCCGTAATTCC-3′)
		TAReUkREV3 (5′-ACTTTCGTTCTTTGATYRA-3′)

## Data Availability

The bacterial, fungal, and protist datasets supporting our study in this manuscript can be found in NCBI repositories. The accession number are PRJNA1187826.
